# Lignins from Agroindustrial by-Products as Natural Ingredients for Cosmetics: Chemical Structure and In Vitro Sunscreen and Cytotoxic Activities

**DOI:** 10.3390/molecules25051131

**Published:** 2020-03-03

**Authors:** Oihana Gordobil, Paula Olaizola, Jesus M. Banales, Jalel Labidi

**Affiliations:** 1 Chemical and Environmental Engineering Department, University of the Basque Country (UPV/EHU), Plaza Europa, 1, 20018 Donostia-San Sebastián, Spain; jalel.labidi@ehu.eus; 2 Department of Liver and Gastrointestinal Diseases, Biodonostia Health Research Institute-Donostia University Hospital, University of the Basque Country (UPV/EHU), 20014 Donostia-San Sebastián, Spain; paula.olaizola@biodonostia.org (P.O.); jesus.banales@biodonostia.org (J.M.B.); 3 Centre for the Study of Liver and Gastrointestinal Diseases (CIBERehd), Carlos III National Institute of Health, 28029 Madrid, Spain; 4 IKERBASQUE, Basque Foundation for Science, 48013 Bilbao, Spain

**Keywords:** hazelnut lignin, walnut lignin, monomeric composition, UV protection, antioxidant, cytotoxicity

## Abstract

The growing concern about the environmental impact and human health risk related to the excessive use of synthetic ingredients in cosmetics and topical formulations calls for the exploration of safe and sustainable natural alternatives. Lignin-rich lignocellulosic industrial wastes such as hazelnut and walnut shells were used as a lignin polymer source. Agro-derived lignins were evaluated as a potential natural active ingredient for health care products. Aside from the structural characteristics of isolated lignins, which were identified by GPC, Py-GC–MS, and 2D HSQC NMR techniques, functional properties such as antioxidant power and UV absorption ability were investigated. The SPF values found for creams containing 5% of hazelnut and walnut lignin content were 6.9 and 4.5, respectively. Additionally, both lignin types presented appropriate protection against UVA radiation, highly interesting property to block the full ultraviolet spectrum. The biological activity of isolated lignins assessed at different concentrations (0.01–1 mg/mL) and different times (24, 48, and 72 h) on murine fibroblast cell line 3T3 suggested their suitability for cosmetic applications.

## 1. Introduction

Growing awareness of the damaging effect of solar UV radiation on human skin has led to the development of numerous organic UV filters since 1930. These synthetic compounds are usually low molecular weight aromatic systems conjugated with carbon-carbon double bonds and carbonyl moieties [1]. At present, these chemical ingredients are incorporated into almost all commercialized cosmetic and personal care products such as creams, make-up and body/hair washes [2]. Furthermore, organic UV filters are widely used as industrial additives in polymers, paints, and textiles.

Recently, the environmental impact related to the excessive consumption of these emerging pollutants is being a matter of concern. Several research works have confirmed the widespread presence of organic UV filters in aquatic ecosystems, such as urban underground water, river, lakes, and the sea [3,4,5,6,7], coming from recreational activities as well as the inefficiency of wastewater treatments plants to eliminate them. Moreover, relevant concentration levels of several chemical sunscreens were detected in aquatic biota, like fish, mussels and marine mammals [8,9,10], evidencing a marked tendency to accumulate in living organisms.

Furthermore, human health risk associated with these hazardous chemicals is another issue of great concern. Humans are daily exposed to organic UV filters, which are capable of reaching the organism not only by percutaneous absorption but also by contaminated food and water consumption. Their bioaccumulation in human fluids, such as urine, semen, breast milk, and the bloodstream [11,12,13], have been recently reported. Although the real effect of these molecules in the human organism is still uncertain, it is well known that some commercialized chemical UV filters exhibit hormonal activity, negatively influencing on the reproduction cycle of organisms [7,14]. Additionally, in recent times, the maternal transfer of organic UV filters in humans [15], dolphins [16], and birds [17] has been proven.

Regarding the regulation of organic sunscreen products, it greatly differs between countries. At present, in Europe, 25 organic UV filters are regulated as cosmetic ingredients by the European Commission (Regulation (EC) Nº 1223/2009, Annex VI). However, the regulation in the US is more stringent. FDA (Food & Drug Administration) treats chemical UV filters as over the counter (OTC) drugs and currently, the use of only 14 organic sunscreens is allowed [18].

As a result, there is high interest in the study of alternatives that could totally or partially replace current synthetic compounds. All this adds up to the increasingly demand of consumers for petrochemical-free products. However, even though natural active ingredients, such as polyphenols are being considered for cosmetic and topical formulations because of their functional properties [2], it is still insufficient scientific work to ensure their no toxicity and pollutant character.

In this context, lignin, as the most abundant polyphenolic compound present in nature, could be an attractive compound to be considered as a bio-active ingredient for cosmetic and personal care products. The aromatic structure of lignin, which is based on phenylpropane units linked through ether and carbon-carbon bonds, contains a wide variety of functional groups capable of absorbing UV radiation (carbon-carbon double bond, carbonyl, and others chromophores), making it a suitable candidate as a broad-spectrum bio-sunscreen agent [19]. The effectiveness of lignin as a sunscreen agent was first evidenced by Qian and co-workers, proving a significant enhancement of the sun protection factor of a commercial sunscreen formulation by the incorporation of lignin in addition to the improvement of the sunscreen performance after UV irradiation [19]. Another remarkable aspect of lignin is its antioxidant capacity. Lignin is able to reduce the formation of free radicals [20], which can be relevant for cosmetic applications. Moreover, the biological activity (antitumor, antiviral, antimicrobial) of lignin has been recently reported [21] exhibiting a promising future for pharmacological and biomedical applications. However, due to the great variety of lignin types together with their complex structure, today it is challenging to provide a defined biological profile for lignin polymer. To date, few studies have been reported about the cytotoxic effect of lignin [22,23], which is totally necessary for ensuring its applicability in human care fields.

Therefore, the present study contributes to the exploration of new natural alternatives to commonly used commercial antioxidants and organic UV filters, promoting the circular bioeconomy by the revalorization of agro-food industrial residues. The industrial activity related to hazelnut and walnut crops generates a large number of shells as by-product every year. Moreover, the world production of these agricultural crops has markedly increased in the last 30 years, making them attractive feedstock to obtain high-value-added products. Currently, these types of solid residues are mainly used for low-value applications such as solid fuel, despising the high lignin content in their chemical composition (30%–50%) [24].

Hence, the high potential of agroindustrial residues such as nut shells as a lignin source together with the lack of characterization of this kind of lignins in comparison to technical lignins and lignins coming from other agricultural wastes like sugarcane bagasse, what straw, sisal, hemp and corn residues [25,26,27] has motivated this research study. In this work, lignin samples were isolated from hazelnut and walnut shells using an ethanol organosolv treatment in order to be evaluated as a natural antioxidant and UV blocker agent. Firstly, the chemical composition as well as the main molecular characteristics such as molecular weight distribution, monomeric composition and the main inter-unit linkages of lignin polymer were analyzed. In addition, the antioxidant capacity and the in vitro sun protection properties were determined. Finally, the cytotoxicity of isolated lignins against mouse fibroblast cell line 3T3 was studied in order to ensure the potential application of lignins from agro-industrial residues in cosmetic and pharmaceutical applications.

## 2. Results and Discussion

### 2.1. Chemical Composition and Molecular Properties of Lignins from Shells

Aside from the origin, the isolation process has a great influence on the lignin quality and chemical structure [28] and therefore other isolation conditions could highly influence on the extraction yields as well as physico-chemical properties of extracted lignins. However, organosolv pulping has been considered as an efficient and environmentally acceptable sulfur-free treatment for the conversion of lignocellulosic residues allowing the obtention of high quality and purity lignins [29,30]. In this work, the yields of lignin achieved from hazelnut and walnut shells were 19.2 ± 0.4 and 15.3 ± 0.1 g/100 g of raw material, respectively. The purity of isolated lignins from nutshells, which was based on lignin content (Klason lignin and acid-soluble lignin), ash and carbohydrates, is reported in Table 1. Lignins from hazelnut (HL) and walnut (WL) shells showed a high purity level, especially in the case of hazelnut lignin, with values of lignin content between 85%–95% and 2%–3% of carbohydrates. Under the same isolation process, HL presented higher Klason lignin content and less carbohydrate contamination than WL. In addition, very low ash content was observed in both evaluated lignins (<0.5%) in comparison with industrial kraft lignins and lignins isolated from agricultural residues such as apple tree pruning, olive tree pruning, and almond shell [31,32]. Although the isolation process was sulfur-free, sulfuric acid was used for the lignin precipitation step, but the elemental analysis confirmed no sulfur contamination because of the precipitation. Regarding the molecular size of agro-waste derived lignins, similar molecular weight distributions were found for both lignins. The polydispersity index (PDI), which reflects the heterogeneity of the lignin polymer, is highly influenced by the origin and isolation process employed for lignin extraction [33]. In this case, ethanol concentration (70%), temperature (180 °C) and selected time (90 min) provided lignins with higher molecular weight and polydispersities than those found for industrial softwood and hardwood kraft lignins [32,34,35], but similar to lignins isolated from other agricultural residues [36].

In addition, Py-GC–MS and 2D HSQC NMR analytical techniques were used in order to elucidate some of the most important chemical characteristics, such as the presence of functional groups and monomeric composition of lignins. The nature of lignin is widely related to the volatile phenolic compounds released from fast pyrolysis [37,38,39,40] which can be grouped into four categories according to their aromatic structure and origin: phenol-type compounds (H), guaiacyl-type compounds (G), syringol-type compounds (S), and catechol-type compounds (Ca). The analytical pyrolysis of lignins from agroindustrial residues was performed at 600 °C, optimal temperature to reach a maximum yield of phenolic monomers and to determine the monomeric composition. In the pyrogram of hazelnut and walnut lignins, around 30 phenolic compounds were identified. Other compounds, such as furan derivatives and long-chain carboxylic acids, were also observed in trace amounts (<2%). The relative content of the identified aromatic monomers was calculated by the sum from the peak areas considering the total peak area as 100%. Table 2 summarizes the recollected information from Py-GC-MS analysis. Unexpectedly, a significant difference in the pyrolysis product distribution was observed for analyzed agro-derived lignins, which evidenced totally different monomeric compositions. In the case of HL, the main released products were guaiacol, 4-methylguaiacol, 4-vinylguaiacol, and isoeugenol. However, syringol, 4-methylsyringol, 4-allysyringol, 3-methoxycatechol and 4-methylguaiacol, were the most representative phenolic compounds found for WL. As can be observed in Table 2, HL was mainly based on guaiacyl units with very small amount of syringyl units, while WL presented more similar monomeric composition to those found for hardwood lignins [41], with 53.2% of syringyl derivatives and 32.2% of guaiacyl-derived compounds detected in the pyrogram. Although syringyl and guaiacyl-type compounds were dominant in both pyrogram, accounting more than 85% of the total peaks, phenol-type and catechol-type compounds were also detected. These compounds are usually produced from demethoxylation (Ph-OCH_3_ cracking) and demethylation (PhO-CH_3_ cracking) of S-type phenols generating phenol derivatives and catechol-type compounds, respectively [37,42]. The appearance of H-type compounds in the pyrogram of WL was substantially higher than found in HL, indicating the presence of p-hydroxyphenyl units (H) as an elemental unit of the walnut lignin structure. Nevertheless, the pyrolysis technique cannot provide a clear conclusion about the origin of the released phenol derivatives. Therefore, the syringyl/guaiacyl ratio (S/G) was calculated by dividing the sum of peak areas from syringyl units (including catechol derivatives) by the sum from the peak areas of guaiacyl derivatives (Table 2). Despite there are numerous research studies regarding the valorization of these biomass wastes, especially for energetic applications, their utilization as a lignin source has been hardly studied. Therefore, there is not a great deal of information regarding the monomeric composition of lignin from agroindustrial residues such as nutshells. Last year, Queiros and coworkers [43] investigated the monomeric composition of lignins from walnut, almond and pine nut shell using analytical pyrolysis and reported S/G ratios of 1.6, 1, and 0, respectively. Other authors studied the relative monomer content of lignin from coconut shells obtaining values of S/G ratio of 0.2–0.3 [44]. However, no prior reports of hazelnut shells delignification were found in literature and consequently, it was not possible to compare our results with previous studies. Furthermore, the characteristics of lignin-derived aromatic products were analyzed by sorting them regarding the structural features of their side chain. The most significant difference between HL and WL was related to the number of phenolic compounds with a side chain attached to the aromatic ring. The content of phenolic compounds with short and long side chains was found higher for HL than for WL. Moreover, the ratio between phenols with short and long side chains clearly showed significant structural differences between isolated lignins. Additionally, aromatic compounds with unsaturated side chains such as vinylsyrirngol, vinylguaiacol, and isoeugenol were more abundant in the case of HL accounting for around 29% of the total released pyrolysis products. High unsaturation levels could have a positive influence on the sunscreen performance of lignin [45]. Although double C=C bonds can be part of the lignin structure as well as being formed during the pyrolysis process [46], oxidized substructures come from the original chemical structure when the lignin is pyrolyzed [39]. Higher content of phenolic compounds with oxygenated groups in the side chains such as aldehyde and ketone functional groups were observed for WL. Therefore, WL has a higher content of carbonyl groups in its chemical structure than HL.

**Figure 1:**
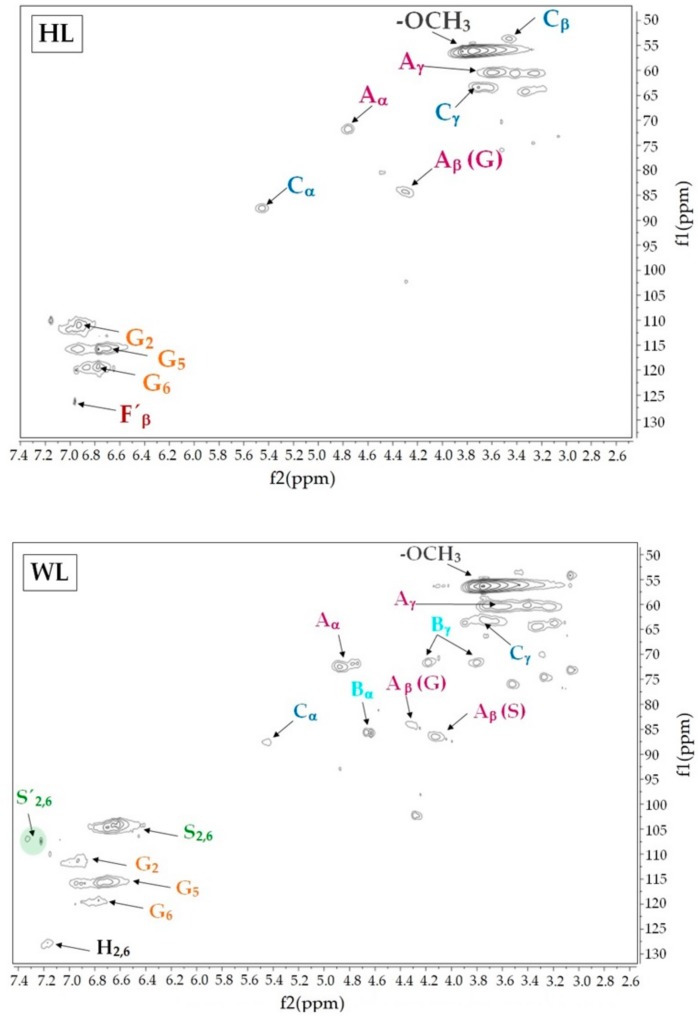
Side chain and aromatic regions of 2D HSQC NMR spectra of isolated lignins.

The HSQC spectra of hazelnut and walnut lignins provided detailed information about their chemical structure and linkages between elemental units. Figure 1 shows the side chain region (δ_C_/δ_H_ 50–90/2.6–6) and the aromatic/unsaturated region (δ_C_/δ_H_ 100–150/5.5–8) of the spectra. The signals assignments reported in Table 3 were determined according to previously reported data [47,48]. In the aromatic region of walnut lignin, cross signals related to syringyl (S_2,6_), C_α_-oxidized syringyl units (S’_2,6_), guaiacyl (G_2_, G_5_ and G_6_) and p-hydroxyphenyl elemental units (H) were observed. This result confirmed the presence of H units in WL and explained the high phenol derivatives content found in the released products during the pyrolysis analysis. Regarding HL, the prominent signals at δ_C_/δ_H_ 119.20/6.77, δ_C_/δ_H_ 115.78/6.77 and δ_C_/δ_H_ 111.00/6.93 associated to C_6_–H_6_, C_5_–H_5_, C_2_–H_2_ in guaiacyl units confirmed the guaiacyl-based chemical structure of this type of lignin. Moreover, a weak signal attributed to C_β_–H_β_ of cynnamyl acetate substructures was also detected in the aromatic region. These substructures could be the origin of the syringyl and catechol derivatives during the pyrolysis of HL. The aliphatic-oxygenated region of the spectra gives information about the different inter-unit linkages present in the lignin structure [49]. The main correlation signals detected in the phenylpropanoid side chain region of HL were associated with β-aryl-ether linkages (β-O-4′) and β-5′ bonds in phenylcoumaran substructures. The results of the side chain of WL, aside from β-aryl-ether linkage (β-O-4′) and β-5′ in phenylcoumaran substructures, β-O-4′ linkages with γ–acetyl groups and resinol substructures formed by β-β´ were also identified. The relative abundance of the main inter-unit linkages was calculated according to Wen et al., 2013 [50] following the proposed semi-quantitative method which uses integral values of the side chain region. The results are represented in Table 4.

### 2.2. Total Phenolic Content (TPC) and Antioxidant Properties of Lignin from Shells

The results of the Folin–Ciocalteau assay and the antioxidant capacity against ABTS and DPPH free radicals were included in Table 5. HL presented significant higher phenolic content than WL with values of 498.0 ± 23.7 μg GAE/mg dry lignin and 281.5 ± 12.1 μg GAE/mg dry lignin, respectively. Although no previous information about the phenolic content of lignin from hazelnut and walnut agro-residues was reported, the results of TPC of studied lignins were in agreement with those found for other lignin types [22,46]. Moreover, with the aim of evaluating the inhibitory effect of isolated lignin samples, BHT (butylated hydroxytoluene) and ascorbic acid were used as controls for the antioxidant assays and the results were expressed as the concentration required for 50% inhibition of the radical (Efficient Concentration value, IC_50_). It has been previously demonstrated that molecular weight distribution, structural characteristics such as functional groups and impurities content of lignin have an important effect on the antioxidant performance [22,51,52,53]. In this study, aside from the clear structural differences between isolated lignins, similar antioxidant power was observed for both agro-derived lignins. Additionally, the results were compared to two commercial antioxidants such as ascorbic acid, more known as Vitamin C, and butylated hydroxytoluene. These antioxidants are two of the most common antioxidant compounds used in the European Union (EU) for food, cosmetic and pharmaceutical applications. However, long-term exposure to high doses of BHT could have negative effects on human health as well as on the environment [54]. Hazelnut and walnut lignins presented lower antioxidant power than ascorbic acid, while the DPPH and ABTS free radical scavenging activity were found quite similar to BHT, concluding that isolated lignins could be a promising bioactive compound to promote the replacement of BHT in several fields. However, to date, the lack of information about the biological activity of lignin polymer in contact with human skin limits its exploitation as an additive in human care products. Consequently, a safety assessment of lignin macromolecule is crucial to develop its biological profile and to ensure the safe application of lignin polymer in cosmetic and topical formulations.

### 2.3. Cytotoxic Properties

**Figure 2:**
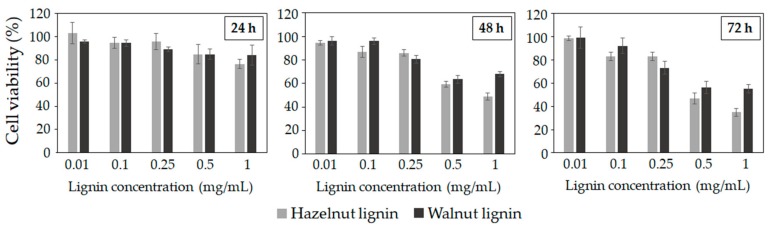
Cell viability (%) of murine fibroblast cell line at different concentrations of lignin at 24 h, 48 h and 72 h of exposure.

**Figure 3:**
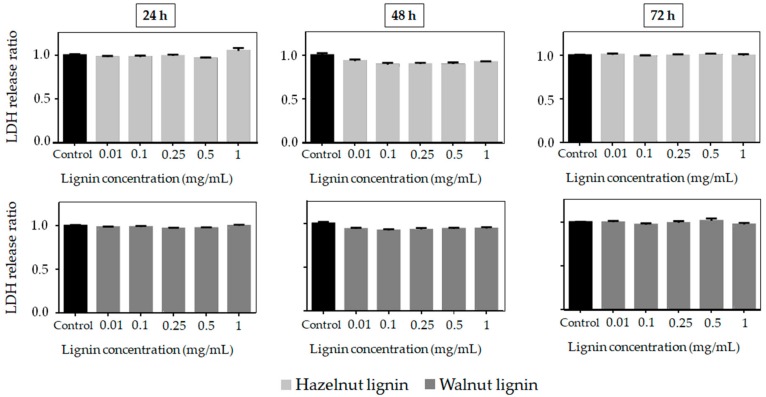
Cell death ratio (based on LDH enzyme release measurement) of murine fibroblast cell line at different concentrations of lignin at 24 h, 48 h and 72 h of exposure.

The potential of lignins as a natural ingredient for cosmetic and pharmaceutical applications makes vital the evaluation of their cytotoxicity. Cell viability and cell death tests are validated in vitro biological methods to predict the toxicity of a wide range of substances [23]. This study shows for the first time the cytotoxic activity of lignins isolated from hazelnut and walnut shells towards the mouse fibroblast cell line 3T3. The cell viability and cell death after lignin addition at different concentrations (0.01–1 mg/mL) and different times (24h, 48h and 72h) are presented in Figure 2 and Figure 3. The cytostatic effect of hazelnut and walnut lignins was concentration and exposure time-dependent. Low concentrations of lignin (0.01–0.1) did not decrease the cell viability, particularly at 24 h and 48 h of exposure. The cell viability at 0.5% of lignin concentration was 85%, indicating that the cells were substantially not affected. Nevertheless, prolonged treatment and high concentrations (0.5% and 1%) had a negative effect on cell proliferation. Interestingly, neither of the lignins incremented cell death even at the highest concentrations or after longer exposure times (Figure 3). Therefore, the lignins could be affecting cell viability through a reduction in cell proliferation, rather than by inducing cytotoxic death. Regarding the lignin type, HL presented more cytostatic activity against mouse fibroblast cells than WL, as observed by the reduction in their viability, especially at high concentrations and long times of treatment. The surviving fraction at 24, 48, and 72 h for HL were 76%, 48% and 34% respectively, while the surviving fraction after WL treatment was 84% (24 h), 67% (48 h), and 55% (72 h).

### 2.4. Sun Protection Properties

**Figure 4:**
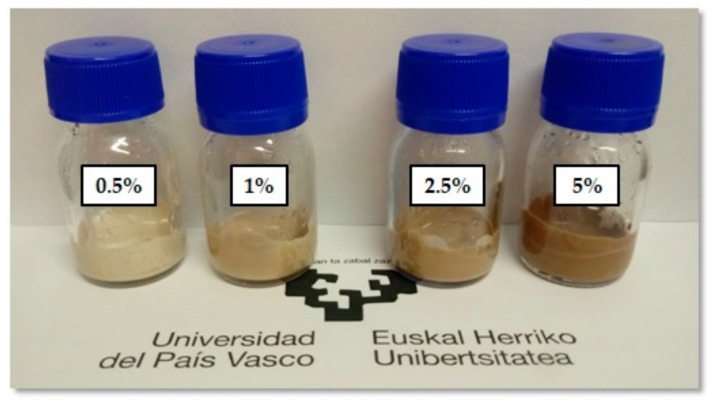
Photograph of the cream with hazelnut lignin addition in different concentrations.

The phenolic nature of lignin with several UV absorbing functional groups makes it especially attractive as a natural ingredient for cosmetics. However, lignin polymer is still insufficiently studied in this field. Therefore, in this work, the UV protection capacity of isolated lignins from hazelnut and walnut shells was evaluated according to the in vitro SPF method based on UV spectrophotometric measurements [55]. Isolated lignin samples were blended with commercial cream at different concentrations (0.5–5%) (Figure 4) and the SPF values, UVA/UVB ratio, and critical wavelength were determined and presented in Table 6. The results demonstrated an increase in the UV absorbance when lignin amount increased into the pure cream, indicating their ability to act as UV absorber compounds. The UV-absorbing property of lignin polymer is due to its chemical structure with chromophores such as carbon-carbon double bonds, carbonyl groups, and aromatic systems. Moreover, auxochromes like hydroxyls groups and ethers play an important role in the sunblock action of lignin [19,56]. The SPF values obtained from lignin-based creams were not high, ranging from 1.4 to 6.5 in the case of HL and 1.3–4.5 for WL. The higher SPF provided by HL could be due to its molecular features such as a (a) lower molecular weight and polydispersity, (b) higher content of unsaturated side chains, and (c) higher total phenolic content than WL [57,58]. These results were in agreement with previously reported studies. Qian and coworkers [19] reported the evaluation of alkali commercial lignin for the development of high-performance broad-spectrum sunscreens, showing maximum SPF of 5.7 at 10% of lignin incorporation to the pure cream. Later, sunscreen performance of lignin from different technical resources was reported with maximum SPF value 8.6 at 10 % for commercial organosolv lignin (chemical point) [45]. However, the maximum SPF values reached with lignin polymer is still insufficient to provide effective sun care according to the current requirements, which consider broad-spectrum sunscreens with SPF values of at least 15 suitable for prevention of sun radiation damage. Nevertheless, one of the main advantages of lignin compared to some commercial organic filters and other natural phenolic compounds like extracts are its ability to block the full UV spectrum light. In this work, the broad-spectrum protection capacity of isolated lignins was demonstrated by the critical wavelength value, defined as the wavelength at which the integral of the spectral absorbance curve reached 90% of the integral from 290 to 400 nm. Therefore, optimal UVA protection can be considered when C_λ_ > 370. Another important parameter related to the effectiveness of the product against UVA radiation is the UVA/UVB ratio [59]. As can be observed both lignin types showed good and superior UVA protection according to the Boost Star Label System [60] with ratios higher than 0.4 and critical wavelength values higher than 370. However, it can be appreciated a clear higher protection capacity of walnut lignin against UVA radiation than found for lignin isolated from hazelnut shells. This result could be explained by the higher content of methoxyl groups present in walnut lignin. Although methoxyl groups are not considered chromophores, they are an important electron-donating group that has the capacity to modify the ability of chromophores to absorb towards long wavelengths in the UV region, as was demonstrated by Qian et al., 2016 [45]. These results suggest that lignin polymer is valuable for UV protection applications in cosmetics, but is still necessary for the development of new technologies to improve the lignin UV protection performance increasing the SPF.

## 3. Materials and Methods

### 3.1. Materials

N,NDimethylformamide (99%), Folin-Cioucalteu phenol reagent, and ascorbic acid were provided by Fisher. Ethanol absolute (99.9%) and gallic acid monohydrate were supplied by Scharlau. Sulfuric acid (96% technical grade), dimethyl sulfoxide (DMSO) and lithium bromide used were from Panreac. 2,2-Diphenyl-1-picrylhydrazyl (DPPH), 2,2´-azino-bis3-ethylbenzothiazoline-6-sulphonic acid (ABTS), butylated hydroxytoluene (BHT) and sodium carbonate were purchased from Sigma-Aldrich.

### 3.2. Lignin Isolation from Nutshells

Hazelnut and walnut shells used as lignin sources were gathered in the Basque Country (Spain). Firstly, the shells were dried and milled to obtain 0.25–0.40 mm particles. Lignin isolation was carried out using an ethanol/water organosolv treatment described in previous work [61]. After the cooking step, the liquid fraction (where lignin was dissolved) was separated from the solid fraction by filtration. Dissolved lignin was isolated by precipitation with two acidified portions of an aqueous solution (pH around 2). The precipitated lignin was filtered, washed until neutral pH and dried at 50 °C. Lignins from hazelnut and walnut shells were called HL and WL, respectively. Isolated lignins were used without further purification.

### 3.3. Chemical Composition

The purity of lignins was assessed in terms of Klason lignin, acid-soluble lignin (ASL), and carbohydrate content, according to the procedure detailed in previous work [32]. The ash content was evaluated using a standard procedure (TAPPI T211 om-02) [62].

### 3.4. Structural Properties

#### 3.4.1. Gel Permeation–High-Performance Liquid Chromatography (GPC)

The molecular weight distribution of isolated lignins was determined by gel permeation chromatography (GPC) on a Jasco LC-Net II/ADC instrument, equipped with a RI-2031 Plus Intelligent refractive index detector, PolarGel-M column (300 mm 7.5 mm) and PolarGel-M guard (50 mm 7.5 mm). 0.25 mg of lignin sample was dissolved in 5mL of N,N-dimethylformamide (DMF) with 0.1% of lithium bromide and 20 μL of solution were injected. The column operated at 40 °C and eluted with N,N-dimethylformamide (DMF) with 0.1% of lithium bromide at a flow of 0.7 mL/min. Polystyrene was used as a standard.

#### 3.4.2. Analytical Pyrolysis

Moreover, the monomeric composition and S/G ratio were estimated by Pyrolysis gas chromatography mass spectrometry (Py-GC-MS), equipped with a 5150 Pyroprobe pyrolyzer (CDS Analytical Inc., Oxford, PA) and GC-MS instrument (Agilent Techs. Inc. 6890 GC/5973 MSD). A quantity between 400–800 mg was pyrolyzed in a quartz capillary tube at 600 °C for 15 s with a heating rate of 20 °C/ms (ramp-off) with the interface kept at 260 °C. The pyrolyzates were purged from the pyrolysis interface into the GC injector under inert conditions using helium gas. The fused-silica capillary column used was an Equity-1701(30 m × 0.20 mm × 0.25 μm). The GC oven program started at 50 °C and was held for 2 min. Then, it was raised to 120 °C at 10 °C/min and was held for 5 min after that raised to 280 °C at 10 °C/min and was held for 8 min and finally raised to 300 °C at 10 C/min and was held for 10 min. The compounds were identified by comparing their mass spectra with the National Institute of Standards Library (NIST) and with compounds reported in the literature [63,64,65].

#### 3.4.3. ^1^H-^13^C HSQC NMR

For the 2D HSQC NMR, around 50 mg of lignin was dissolved in 0.5 mL of DMSO-d6. 2D HSQC NMR spectra were recorded at 25 °C in a Bruker AVANCE 500 MHz equipped with a z-gradient double resonance probe. The spectral widths for the HSQC were 5000 and 12300 Hz for the ^1^H and ^13^C dimensions, respectively. The number of collected complex points was 1024 for the ^1^H dimension with a recycle delay of 1.5 s. The number of transients was 64, and 256 time increments were always recorded in the ^13^C dimension. The ^1^J*_CH_* used was 145 Hz. Prior to Fourier transformation, the data matrices were zero filled to 2048 points in the ^13^C dimension. Data processing was performed using MestReNova software. The central solvent (DMSO) peak was used as an internal chemical shift reference point (δ_C_/δ_H_ 39.5/2.49).

### 3.5. Total Phenolic Content (TPC) and Antioxidant Activity

The TPC of isolated lignins was determined by the Folin–Ciocalteau spectrophotometric method using gallic acid as reference and dimethyl sulfoxide as a solvent, in a Jasco V-630 spectrophotometer equipment. For the analysis, 0.5 mL of lignin solution (2 mg/mL), 2.5 mL of Folin–Ciolcalteau reagent and 5 mL of Na_2_CO_3_ (200 mg/mL) were added to 50 mL flask and covered with distilled water. The samples were kept in a thermostatic bath at 40 °C for 30 min before the spectrophotometric measurement of the absorbance at 750 nm. The total phenolic content of lignin samples was expressed as μg gallic acid (GAE) per mg of dry lignin. All parameters were calculated on a dry basis. The antioxidant activity of isolated lignins was evaluated by DPPH (2,2-Diphenyl-1-picrylhydrazyl) and ABTS (2,2´-azino-bis3-ethylbenzothiazoline-6-sulphonic acid) methods. The DPPH scavenging activity was assessed according to the method described by Brand-Williams et al., 1995 [66] with some modifications. DMSO was also used to dissolve the lignin samples at different concentrations (0–2 mg/mL). 0.1 mL of lignin solution was added to 3.9 mL DPPH (25 mg/L in methanol). The absorbance was measured at 517 nm after 30 min of incubation at room temperature. For the ABTS assay, a radical solution was prepared by the reaction of 7 mM ABTS and 2.45 mM potassium persulfate. The radical solution was left to stand in the dark at room temperature for 16 h before using it. The ABTS solution was diluted with ethanol to reach an absorbance of 0.70 ± 0.02 at 734 nm. For the analysis, 2 mL of the diluted radical solution was mixed with 20 μL of the sample and the absorbance at 734 nm was read after 6 min. Commercial antioxidants (BHT and ascorbic acid) were used as a positive control. Each test was carried out in triplicate. The radical scavenging activity of the lignin was expressed using the term “efficient concentration” or IC_50_, which is the concentration required for 50% inhibition of the free radical.

### 3.6. Cytotoxicity Test

Cell viability was assessed using Cell Proliferation WST-1 Assay (Roche) according to the manufacturer’s instructions. Briefly, 3T3 mouse fibroblasts were seeded in 96-well plates (1500, 3000 and 5000 cells per well for 24, 48 and 72 h, respectively) in DMEM supplemented with 10% fetal bovine serum and 1% penicillin/streptomycin and incubated O/N at 37 °C in a 5% CO_2_ atmosphere. Then, the cells in culture were exposed to increasing concentrations (from 0.01 to 1 mg/mL) of lignins (HL and WL) using DMSO as a vehicle for 24, 48 and 72 h. Vehicle-treated cells were considered the control group. Finally, 10 μL of WST-1 (cell proliferation reagent) were added to each well, incubated at 37 °C for 1 h and absorbance was measured at 450 nm in a Halo LED 96 microplate reader (Dynamica). The cell viability of the treated cells was calculated relative to the control group. In addition, cell death was evaluated using the Cytotoxicity Detection KitPLUS, LDH (lactate dehydrogenase) (Roche) according to manufacturer’s instructions. The protocol followed to culture and seed the cells was the same as carried out for the cell viability test. After the incubation, the cells in culture were exposed to lignin samples at the same concentrations and time established for the cell viability assay. In the end, the reaction mixture was added (50 µL to each well), incubated for 30min at 37 °C in the dark, subsequently, the absorbance measurements were made at 492 nm in a Halo LED 96 microplate reader (Dynamica).

### 3.7. Preparation and Evaluation of Lignin Sunscreen Properties

Lignin-based sunscreen samples were prepared by blending isolated lignins from shells with the pure cream (NIVEA body milk) at different concentrations (0.5–5%) and stirring under room temperature for 48 h in the dark to obtain a homogeneous dispersion. NIVEA SPF 30 was used as positive control and NIVEA body milk cream was taken as a negative control. The measurements of sun protection parameters of isolated lignins were performed according to the protocol proposed by Diffey and Robson., 1989 [55], which allows a rapid assessment of the sun protection factor (SPF) as well as the study of the product against long-wavelength ultraviolet radiation. Before UV transmittance measurements, lignin-based sunscreen samples were applied at 2 mg/cm^2^ to 3M Transpore^®^ Tape (solid substrate) and then dried in the dark for 15–20 min. UV transmittance of lignin-based sunscreens was measured in a Jasco V-630 spectrophotometer from 290 to 400 nm per 0.5 nm. The collected transmittance spectra were used to calculate the in vitro sun protection factor (SPF) (Equation (1)).

(1) $$\mathrm{SPF}=\frac{\sum_{290}^{400}E_{\lambda }B_{\lambda }}{\sum_{290}^{400}\frac{E_{\lambda }B_{\lambda }}{{\mathrm{MPF}\lambda }_{}}}$$

where E_λ_ denotes CIE erythemal spectral effectiveness, B_λ_ is olar spectral irradiance, and MPFλ is spectral transmittance of the solid substrate/ spectral transmittance of the sample. In addition, to evaluate the efficiency of the lignins protection against UVA radiation, UVA/UVB ratio and the critical wavelength (C_λ_) were calculated from the absorbance spectrum using Equations (2) and (3), respectively.

(2) UVAUVBratio=∑320400AλΔλ∑320400Δλ∑280320AλΔλ∑280320Δλ

(3) $$C\lambda =\int_{280}^{C\lambda }A_{\lambda }d\lambda =0.9\int_{280}^{400}A_{\lambda }d\lambda$$

## 4. Conclusions

In this work, lignins from hazelnut and walnut shells were evaluated as potential bioactive ingredients for cosmetic products. Aside from the structural characterization, which was reported for the first time in the present study, the antioxidant properties and sunscreen capacity of isolated lignins were studied. Moreover, as an essential requirement for cosmetics and topical formulations, biological activity towards murine fibroblast cells line 3T3 was examined. Isolation conditions used resulted in high pure lignins (>85%) with low carbohydrate and very low ash content. Regarding their molecular characteristics, significant structural differences were found between both agro-derived lignin polymers. HL presented a guaiacyl units-based condensed chemical structure rich in unsaturated functional groups in the side chain of the aromatic units, while WL showed a chemical structure with high content of syringyl units and the presence of guaiacyl and traces of *p*-hydroxyphenyl units. Additionally, the presence of carbonyl functional groups in WL was associated with its partially acylated side chain and oxidized syringyl units. Concerning their potential as a bio-active ingredient for cosmetics, a suitable antioxidant activity and comparable to BHT commercial antioxidant was found for both lignin types. Moreover, the biological analyses demonstrated that agro-derived lignins exhibited a concentration and exposure time-dependent cytostatic effect on 3T3 cell line, which was stronger in the case of HL. Nevertheless, it was observed that cells were not substantially affected by the incorporation of lignins at 24 h of exposure, indicating no cytotoxic behavior. In addition, isolated lignins presented UV-absorbing properties, but the sun protection factor provided by lignin incorporation in pure cream did not reach the current requirements established for the prevention of sun radiation skin damage. Although a strategy to increase the UV performance of lignin polymer is still needed, their absence of significant cytotoxicity opens a promising valorization route for lignin derived from agro-industrial wastes in cosmetic and pharmaceutical fields.

## Figures and Tables

**Table 1:** Chemical composition (dry basis %) and molecular weight distribution of lignins.

	HL	WL
Klason lignin (%)	94.2 ± 0.5	85.7 ± 0.4
ASL (%)	0.8 ± 0.2	3.4 ± 0.3
Carbohydrate content (%)	2.2 ± 0.0	2.7 ± 0.1
Ash content (%)	<0.5	<0.5
S (%)	nd*	nd*
Mn	1613	1545
Mw	9282	9630
PDI	5.7	6.2

*nd: not detected.

**Table 2:** Relative content (%) of phenolic-type compounds and their classification according to their structural characteristics.

Pyrolysis Products	Hazelnut Lignin (HL)	Walnut Lignin (WL)
S-type compounds	5.3	53.2
G-type compounds	83.6	32.2
Ca-type compounds	8.7	7.9
H-type compounds	2.5	6.7
S/G	0.2	1.9
Methoxylated aromatic compounds (%)^1^	88.9	91.6
Non-substituted saturated chains (%)^2^	49.1	45.1
Unsaturated side chains (C_α_=C_β_) (%)	28.4	9.7
Unsaturated side chains (C_β_=C_γ_) (%)	0.5	7.6
Oxygenated groups in the side chains (C=O) (%)	9.7	11.5
Short side chain (C_1_+C_2_)	70.2	63.4
Long side chain (C_3_)	17.0	2.9
(ArC_1_+ArC_2_)/ArC_3_^3^	4.1	21.9

^1^ Guaiacyl and syringyl derived compounds; ^2^ Short and propanoid side chains attached to the aromatic ring; ^3^ Ratio between phenols with short and long side chains.

**Table 3:** Assignments of ^13^C-^1^H cross signals in the HSQC spectra shown in Figure 1.

Label	δC/δH (ppm)	Assignments
H	127.74/7.17	C_2_-H_2_ and C_6_-H_6_ in H units
F´_β_	126.24/6.96	C_β_–H_β_ of cinnamyl acetate end-groups (F´)
G_6_	119.20/6.77	C_6_-H_6_ in guaiacyl units (G)
G_5_	115.78/6.77	C_5_-H_5_ in guaiacyl units (G)
G_2_	111.00/6.98	C_2_-H_2_ in guaiacyl units (G)
S´_2,6_	106.97/7.33	C_2_-H_2_ and C_6_-H_6_ in oxidized S units (S´)
S_2,6_	104.14/6.61	C_2_-H_2_ and C_6_-H_6_ in S units (S)
C_α_	87.58/5.45	C_α_-H_α_ in β-5´ (phenylcoumaran) substructrures (C)
A´_β_ (S)	86.51/4.13	C_β_-H_β_ in γ-acetylated β-O-4´ substructures linked to S unit (A)
B_α_	85.76/4.63	C_α_-H_α_ in β-β´ resinol substructures (B)
A_β_ (G)	84.20/4.31	C_β_-H_β_ in β-O-4´ substructures linked to G unit (A)
A_α_ (G)	71.70/4.76	C_α_-H_α_ in β-O-4´ substructures (A)
B_γ_	71.62/3.80–4.20	C_γ_-H_γ_ in β-β´ resinol substructures (B)
C_γ_	63.28/3.73	C_γ_-H_γ_ in β-5 (phenylcoumaran) substructrures (C)
A_γ_	60.3/3.70	C_γ_–H_γ_ in β-O-4´ substructures (A)
C_β_	53.64/3.46	C_β_-H_β_ in β-5´ (phenylcoumaran) substructrures (C)
-OCH_3_	56.11/3.76	C-H in methoxyl groups

**Table 4:** Relative abundance of the main inter-unit linkages from the integration of ^13^C-^1^H correlation signals in the HSQC spectra.

Linkage Abundance (%)	β-O-4´ (A)	β-O-4´ (A´)	β-β´ (B)	β-5´ (C)
Hazelnut lignin (HL)	54.1	-	-	45.9
Walnut lignin (WL)	44.7	8.3	20.1	26.8

**Table 5:** Total phenolic content (TPC) and antiradical properties of isolated lignins determined by DPPH and ABTS assays.

Samples	TPC	ABTS	DPPH
	μg GAE/mg Dry Lignin	IC_50_ (μg/mL)	IC_50_ (μg/mL)
HL	498.0 ± 23.7	9.74 ± 0.7	20.07 ± 0.3
WL	281.5 ± 12.1	9.63 ± 0.4	19.17 ± 0.5
BHT	-	7.74 ± 0.2	18.96 ± 0.1
Ascorbic acid	-	2.40 ± 0.0	2.93 ± 0.0

**Table 6:** SPF, UVA/UVB ratio and critical wavelength values (C_λ_) of the pure cream blended with hazelnut and walnut lignins.

	Lignin Content (%)	SPF	UVA/UVB	C_λ_
N-HL	0.5	1.4	0.44	374.5
N-HL	1	1.7	0.56	377.0
N-HL	2.5	3.0	0.62	381.5
N-HL	5	6.5	0.62	382.0
N-WL	0.5	1.3	0.56	383.0
N-WL	1	1.6	0.60	383.0
N-WL	2.5	2.0	0.65	384.0
N-WL	5	4.5	0.69	384.5
Control-N	-	0.9	0.23	365.5
Nivea-SPF30	-	28.4	0.76	383.5

## References

[1] Chisvert A., Salvador A., Salvador A., Chisvert A. (2017). Ultraviolet Filters in Cosmetics: Regulatory Aspects and Analytical Methods. Analysis of Cosmetic Products.

[2] Rodrigues F., de la Cádiz-Gurrea L.M., Nunes M.A., Pinto D., Vinha A.F., Linares I.B., Oliveira M.B.P.P., Carretero A.S., Galanakis C. (2018). Cosmetics. Polyphenols: Properties, Recovery, and Applications.

[3] Balmer M.E., Buser H.R., Müller M.D., Poiger T. (2005). Occurrence of some organic UV filters in wastewater, in surface waters, and in fish from Swiss lakes. Environ. Sci. Technol..

[4] Jurado A., Gago-Ferrero P., Vàzquez-Suñé E., Carrera J., Pujades E., Díaz-Cruz M.S., Barceló D. (2014). Urban groundwater contamination by residues of UV filters. J. Hazard. Mater..

[5] Apel C., Joerss H., Ebinghaus R. (2018). Environmental occurrence and hazard of organic UV stabilizers and UV filters in the sediment of European North and Baltic Seas. Chemosphere.

[6] Sánchez Rodríguez A., Rodrigo Sanz M., Betancort Rodríguez J.R. (2015). Occurrence of eight UV filters in beaches of Gran Canaria (Canary Islands). An approach to environmental risk assessment. Chemosphere.

[7] Fent K., Zenker A., Rapp M. (2010). Widespread occurrence of estrogenic UV-filters in aquatic ecosystems in Switzerland. Environ. Pollut..

[8] Gago-Ferrero P., Díaz-Cruz M.S., Barceló D. (2015). UV filters bioaccumulation in fish from Iberian river basins. Sci. Total Environ..

[9] Molins-Delgado D., Muñoz R., Nogueira S., Alonso M.B., Torres J.P., Malm O., Ziolli R.L., Hauser-Davis R.A., Eljarrat E., Barceló D. (2018). Occurrence of organic UV filters and metabolites in lebranche mullet (Mugil liza) from Brazil. Sci. Total Environ..

[10] Gago-Ferrero P., Alonso M.B., Bertozzi C.P., Marigo J., Barbosa L., Cremer M., Secchi E.R., Azevedo A., Lailson-Brito J., Torres J.P.M. (2013). First determination of UV filters in marine mammals. octocrylene levels in Franciscana dolphins. Environ. Sci. Technol..

[11] León Z., Chisvert A., Tarazona I., Salvador A. (2010). Solid-phase extraction liquid chromatography-tandem mass spectrometry analytical method for the determination of 2-hydroxy-4-methoxybenzophenone and its metabolites in both human urine and semen. Anal. Bioanal. Chem..

[12] Molins-Delgado D., del Olmo-Campos M.M., Valeta-Juan G., Pleguezuelos-Hernández V., Barceló D., Díaz-Cruz M.S. (2018). Determination of UV filters in human breast milk using turbulent flow chromatography and babies’ daily intake estimation. Environ. Res..

[13] Matta M.K., Zusterzeel R., Pilli N.R., Patel V., Volpe D.A., Florian J., Oh L., Bashaw E., Zineh I., Sanabria C. (2019). Effect of sunscreen application under maximal use conditions on plasma concentration of sunscreen active ingredients. JAMA.

[14] Weisbrod C.J., Kunz P.Y., Zenker A.K., Fent K. (2007). Effects of the UV filter benzophenone-2 on reproduction in fish. Toxicol. Appl. Pharmacol..

[15] Valle-Sistac J., Molins-Delgado D., Díaz M., Ibáñez L., Barceló D., Silvia Díaz-Cruz M. (2016). Determination of parabens and benzophenone-type UV filters in human placenta: First description of the existence of benzyl paraben and benzophenone-4. Environ. Int..

[16] Alonso M.B., Feo M.L., Corcellas C., Gago-Ferrero P., Bertozzi C.P., Marigo J., Flach L., Meirelles A.C.O., Carvalho V.L., Azevedo A.F. (2015). Toxic heritage: Maternal transfer of pyrethroid insecticides and sunscreen agents in dolphins from Brazil. Environ. Pollut..

[17] Molins-Delgado D., Mánez M., Andreu A., Hiraldo F., Eljarrat E., Barceló D., Díaz-Cruz M.S. (2017). A Potential New Threat to Wild Life: Presence of UV Filters in Bird Eggs from a Preserved Area. Environ. Sci. Technol..

[18] U.S. Food and Drug Administration: Part 352-Sunscreen Drug Products for over-the-Counter Human Use. https://www.accessdata.fda.gov/scripts/cdrh/cfdocs/cfcfr/CFRSearch.cfm?CFRPart=352.

[19] Qian Y., Qiu X., Zhu S. (2015). Lignin: A nature-inspired sun blocker for broadspectrum Sunscreens. Green Chem..

[20] Espinoza-Acosta J.L., Torres-Chávez P.I., Ramírez-Wong B., López-Saiz C.M., Montaño-Leyva B. (2016). Antioxidant, antimicrobial, and antimutagenic properties of technical lignins and their applications. BioResources.

[21] Spiridon I., Poni P., Chemistry M., Ghica G., Alley V. (2018). Biological and pharmaceutical applications of lignin and its derivatives: A Mini-Review. Cellul. Chem. Technol..

[22] Barapatre A., Meena A.S., Mekala S., Das A., Jha H. (2016). In vitro evaluation of antioxidant and cytotoxic activities of lignin fractions extracted from *Acacia nilotica*. Int. J. Biol. Macromol..

[23] Vinardell M.P., Ugartondo V., Mitjans M. (2008). Potential applications of antioxidant lignins from different sources. Ind. Crops Prod..

[24] Demirbaş A. (2005). Estimating of structural composition of wood and non-wood biomass samples. Energy Sources.

[25] Ponomarenko J., Lauberts M., Dizhbite T., Lauberte L., Jurkjane V., Telysheva G. (2015). Antioxidant activity of various lignins and lignin-related phenylpropanoid units with high and low molecular weight. Holzforschung.

[26] Kaur R., Uppal S.K. (2015). Structural characterization and antioxidant activity of lignin from sugarcane bagasse. Colloid Polym. Sci..

[27] Monteil-Rivera F., Phuong M., Ye M., Halasz A., Hawari J. (2013). Isolation and characterization of herbaceous lignins for applications in biomaterials. Ind. Crops Prod..

[28] Kumar A., Anushree K.J., Bhaskar T. (2020). Utilization of lignin: A sustainable and eco-friendly approach. J. Energy Inst..

[29] Weinwurm F., Drljo A., Waldmüller W., Fiala B., Niedermayer J., Friedl A. (2016). Lignin concentration and fractionation from ethanol organosolv liquors by ultra- and nanofiltration. J. Clean. Prod..

[30] Cybulska I., Brudecki G.P., Zembrzuska J., Schmidt J.E., Lopez C.G.B., Thomsen M.H. (2017). Organosolv delignification of agricultural residues (date palm fronds, *Phoenix dactylifera L*.) of the United Arab Emirates. Appl. Energy.

[31] Sequeiros A., Labidi J. (2017). Characterization and determination of the S/G ratio via Py-GC/MS of agricultural and industrial residues. Ind. Crops Prod..

[32] Gordobil O., Moriana R., Zhang L., Labidi J., Sevastyanova O. (2016). Assessment of technical lignins for uses in biofuels and biomaterials: Structure-related properties, proximate analysis and chemical modification. Ind. Crops Prod..

[33] Pan X., Kadla J.F., Ehara K., Gilkes N., Saddler J.N. (2006). Organosolv ethanol lignin from hybrid poplar as a radical scavenger: Relationship between lignin structure, extraction conditions, and antioxidant activity. J. Agric. Food Chem..

[34] Gordobil O., Delucis R., Egüés I., Labidi J. (2015). Kraft lignin as filler in PLA to improve ductility and thermal properties. Ind. Crops Prod..

[35] Jiang X., Savithri D., Du X., Pawar S., Jameel H., Chang H.M., Zhou X. (2017). Fractionation and Characterization of Kraft Lignin by Sequential Precipitation with Various Organic Solvents. ACS Sustain. Chem. Eng..

[36] Rossberg C., Bremer M., Machill S., Koenig S., Kerns G., Boeriu C., Windeisen E., Fischer S. (2015). Separation and characterisation of sulphur-free lignin from different agricultural residues. Ind. Crops Prod..

[37] Jiang G., Nowakowski D.J., Bridgwater A.V. (2010). Effect of the temperature on the composition of lignin pyrolysis products. Energy Fuels.

[38] Shao L., Zhang X., Chen F., Xu F. (2017). Fast pyrolysis of Kraft lignins fractionated by ultrafiltration. J. Anal. Appl. Pyrolysis.

[39] Lin X., Sui S., Tan S., Pittman C., Sun J., Zhang Z. (2015). Fast pyrolysis of four lignins from different isolation processes using Py-GC/MS. Energies.

[40] Constant S., Wienk H.L.J., Frissen A.E., de Peinder P., Boelens R., van Es D.S., Grisel R.J.H., Weckhuysen B.M., Huijgen W.J.J., Gosselink R.J.A. (2016). New insights into the structure and composition of technical lignins: A comparative characterisation study. Green Chem..

[41] Derkacheva O.Y. (2013). Estimation of aromatic structure contents in hardwood lignins from IR absorption spectra. J. Appl. Spectrosc..

[42] Zhao J., Xiuwen W., Hu J., Liu Q., Shen D., Xiao R. (2014). Thermal degradation of softwood lignin and hardwood lignin by TG-FTIR and Py-GC/MS. Polym. Degrad. Stab..

[43] Queirós C.S.G.P., Cardoso S., Lourenço A., Ferreira J., Miranda I., Lourenço M.J.V., Pereira H. (2020). Characterization of walnut, almond, and pine nut shells regarding chemical composition and extract composition. Biomass Convers. Biorefinery.

[44] Avelino F., de Oliveira D.R., Mazzetto S.E., Lomonaco D. (2019). Poly(methyl methacrylate) films reinforced with coconut shell lignin fractions to enhance their UV-blocking, antioxidant and thermo-mechanical properties. Int. J. Biol. Macromol..

[45] Qian Y., Qiu X., Zhu S. (2016). Sunscreen performance of lignin from different technical resources and their general synergistic effect with synthetic sunscreens. ACS Sustain. Chem. Eng..

[46] Ponomarenko J., Dizhbite T., Lauberts M., Viksna A., Dobele G., Bikovens O., Telysheva G. (2014). Characterization of softwood and hardwood lignoboost kraft lignins with emphasis on their antioxidant activity. BioResources.

[47] Rencoret J., Marques G., Gutiérrez A., Nieto L., Santos J.I., Jiménez-Barbero J., Martínez Á.T., Del Río J.C. (2009). HSQC-NMR analysis of lignin in woody (*Eucalyptus globulus* and *Picea abies*) and non-woody (Agave sisalana) ball-milled plant materials at the gel state. Holzforschung.

[48] Yuan T.-Q., Sun S.-N., Xu F., Sun R.-C. (2011). Characterization of lignin structures and LCC linkages by quantitative 13C and 2D HSQC NMR spectroscopy. J. Agric. Food Chem..

[49] Del Río Andrade J.C., Rencoret J., Prinsen P., Martínez Á.T., Gutiérrez Suárez A., Ralph J. (2012). Structural characterization of wheat straw lignin as revealed by analytical pyrolysis, 2D-NMR, and reductive cleavage methods. J. Agric. Food Chem..

[50] Wen J.L., Sun S.L., Xue B.L., Sun R.C. (2013). Recent advances in characterization of lignin polymer by solution-state nuclear magnetic resonance (NMR) methodology. Materials.

[51] Ponomarenko J., Dizhbite T., Lauberts M., Volperts A., Dobele G., Telysheva G. (2015). Analytical pyrolysis-A tool for revealing of lignin structure-antioxidant activity relationship. J. Anal. Appl. Pyrolysis.

[52] Dizhbite T., Telysheva G., Jurkjane V., Viesturs U. (2004). Characterization of the radical scavenging activity of lignins-Natural antioxidants. Bioresour. Technol..

[53] Alzagameem A., El Khaldi-Hansen B., Büchner D., Larkins M., Kamm B., Witzleben S., Schulze M. (2018). Lignocellulosic biomass as source for lignin-based environmentally benign antioxidants. Molecules.

[54] Jayalakshmi C.P., Sharma J.D. (1986). Effect of butylated hydroxyanisole (BHA) and butylated hydroxytoluene (BHT) on rat erythrocytes. Environ. Res..

[55] Diffey B.L., Robson J. (1989). A new substrate to measure sunscreen protection factors throughout the ultraviolet spectrum. J. Soc. Cosmet. Chem..

[56] Gould R.F., Gould R.F. (1966). Lignin Structure and Reactions, Edition 267, Advances in Chemistry Series N° 59.

[57] Glasser G.W., Sarkanen S. (1989). Lignin Structure and Reactions. Am. Chem. Soc..

[58] Toh K., Nakano S., Yokoyama H., Ebe K., Gotoh K., Noda H. (2005). Anti-deterioration effect of lignin as an ultraviolet absorbent in polypropylene and polyethylene. Polym. J..

[59] Gutiérrez-Hernández J.M., Escalante A., Murillo-Vázquez R.N., Delgado E., González F.J., Toríz G. (2016). Use of Agave tequilana-lignin and zinc oxide nanoparticles for skin photoprotection. J. Photochem. Photobiol. B Biol..

[60] Li S.X., Li M.F., Bian J., Wu X.F., Peng F., Ma M.G. (2019). Preparation of organic acid lignin submicrometer particle as a natural broad-spectrum photo-protection agent. Int. J. Biol. Macromol..

[61] Gordobil O., Egüés I., Llano-Ponte R., Labidi J. (2014). Physicochemical properties of PLA lignin blends. Polym. Degrad. Stab..

[62] TAPPI T 211 om-02 (2002). Ash in wood, pulp, paper and paperboard: Combustion at 525 °C. TAPPI test methods.

[63] Wang S., Ru B., Lin H., Sun W., Luo Z. (2015). Pyrolysis behaviors of four lignin polymers isolated from the same pine wood. Bioresour. Technol..

[64] Zhang J., Fleury E., Chen Y., Brook M.A. (2015). Flame retardant lignin-based silicone composites. RSC Adv..

[65] Fernández-Rodríguez J., Gordobil O., Robles E., González-Alriols M., Labidi J. (2017). Lignin valorization from side-streams produced during agricultural waste pulping and total chlorine free bleaching. J. Clean. Prod..

[66] Brand-Williams W., Cuvelier M.E., Berset C. (1995). Use of a free radical method to evaluate antioxidant activity. Leb. Wiss. Technol..

